# The Nallan–Nickel Effect: A Mechanistic Perspective on Burning Sensations and Lichenoid Reactions in Long-Serving Porcelain-Fused-to-Metal Restorations

**DOI:** 10.3390/dj13110507

**Published:** 2025-11-03

**Authors:** Nallan C. S. K. Chaitanya, Nada Tawfig Hashim, Vivek Padmanabhan, Md Sofiqul Islam, Rasha Babiker, Riham Mohammed, Muhammed Mustahsen Rahman

**Affiliations:** 1 Department of Oral Medicine and Radiology, RAK College of Dental Sciences, RAK Medical & Health Sciences University, Ras-AlKhaimah 12973, United Arab Emirates; 2 Department of Periodontics, RAK College of Dental Sciences, RAK Medical & Health Sciences University, Ras-AlKhaimah 12973, United Arab Emirates; 3 Department of Pediatric and Preventive Dentistry, RAK College of Dental Sciences, RAK Medical & Health Sciences University, Ras-AlKhaimah 12973, United Arab Emirates; 4 Department of Operative Dentistry, RAK College of Dental Sciences, RAK Medical and Health Sciences University, Ras-AlKhaimah 12973, United Arab Emirates; sofiqul.islam@rakmhsu.ac.ae; 5 Department of Physiology, RAK College of Medical Sciences, RAK Medical & Health Sciences University, Ras-AlKhaimah 11127, United Arab Emirates; 6 Department of Oral Surgery, RAK College of Dental Sciences, RAK Medical & Health Sciences University, Ras-AlKhaimah 12973, United Arab Emirates

**Keywords:** dental alloys, nickel, corrosion, burning mouth–like symptoms, oral lichenoid contact lesions, TRPV1, TLR4, neurogenic inflammation, galvanic coupling, porcelain-fused-to-metal, Nallan–Nickel effect

## Abstract

Porcelain-fused-to-metal (PFM) crowns continue to serve as a cornerstone of restorative dentistry owing to their strength, affordability, and esthetics. However, late-onset complications such as oral burning and lichenoid reactions have been observed in long-serving PFMs, suggesting complex host–material interactions that extend beyond simple mechanical wear. This Perspective introduces the Nallan–Nickel Effect, a theoretical model proposing that a host- and environment-dependent threshold of bioavailable nickel ions (Ni^2+^), once exceeded, may trigger a neuro-immune cascade culminating in a burning phenotype. Within this framework, slow corrosion at exposed PFM interfaces releases Ni^2+^ into saliva and crevicular fluid, facilitating epithelial uptake and activation of innate immune sensors such as TLR4 and NLRP3. The resulting cytokine milieu (IL-1β, IL-6, TNF-α) drives NF-κB, mediated inflammation and T-cell activation, while neurogenic mediators—including nerve growth factor (NGF), substance P, and CGRP—sensitize TRPV1/TRPA1 nociceptors, establishing feedback loops of persistent burning and neurogenic inflammation. Modifying factors such as low salivary flow, acidic oral pH, mixed-metal galvanic coupling, and parafunctional stress can lower this threshold, whereas replacement with high-noble or all-ceramic materials may restore tolerance. The model generates testable predictions: elevated local free Ni^2+^ levels and increased expression of TLR4 and TRPV1 in symptomatic mucosa, along with clinical improvement following substitution of nickel-containing restorations. Conceptually, the Nallan–Nickel Effect reframes PFM-associated burning and lichenoid lesions as threshold-governed, neuro-immune phenomena rather than nonspecific irritations. By integrating corrosion chemistry, mucosal immunology, and sensory neurobiology, this hypothesis offers a coherent, testable framework for future translational research and patient-centered management of PFM-related complications.

## 1. Background

Porcelain-fused-to-metal (PFM) crowns have long been a benchmark in fixed prosthodontics because they balance durability, esthetics, and affordability [1,2,3,4,5]. While all-ceramic alternatives—especially zirconia—are increasingly popular for translucency and biocompatibility, PFMs remain widely used in cost-sensitive settings [3,4]. Their combination of a strong metallic substructure with an esthetic veneer explains their persistence in routine practice [5].

PFM crowns are supported by a metallic substructure whose composition varies by alloy class [6]. Base-metal systems commonly contain nickel, cobalt, chromium, and molybdenum: nickel adds strength but is the most frequent dental sensitizer [7,8]; cobalt contributes rigidity; chromium forms a protective oxide; and molybdenum improves resistance to pitting [9,10]. Minor elements (iron, manganese, silicon, carbon) fine-tune mechanical performance [11]. Noble and high-noble alloys incorporate greater proportions of precious metals that enhance passivity and biocompatibility (e.g., palladium for hardness/corrosion resistance; gold for corrosion resistance/ductility) [12,13,14,15].

PFM crowns remain one of the most frequently used restorative options worldwide due to their balance of strength, cost-effectiveness, and aesthetics [16,17]. However, their long-term biological impact has not been adequately addressed, particularly the delayed complications arising from gradual porcelain wear and subsequent metal ion release [18]. The emergence of persistent burning sensations and lichenoid reactions in some long-term PFM wearers represents more than a localized adverse effect, it can significantly impair oral function, patient comfort, and overall quality of life [19,20].

This article is presented as a Perspective. It synthesizes relevant mechanistic, clinical, and translational evidence to support the development of the proposed hypothesis, the Nallan–Nickel effect. References were identified through targeted searches in PubMed and Scopus using keywords such as “nickel corrosion,” “porcelain-fused-to-metal complications,” “burning mouth,” “oral lichenoid lesions,” “TLR4/NLRP3,” and “TRPV1 sensitization.” Additional references were selected based on their relevance to neuro-immune mechanisms and dental alloy biocompatibility, complemented by the authors’ expertise in periodontology, prosthodontics, and oral medicine.

Building on emerging mechanistic evidence, we introduce the concept of the Nallan–Nickel effect, a proposed hypothesis that suggests nickel ion release from exposed metallic substructures could reach a critical threshold beyond which mucosal irritation, immune sensitization, and TRPV1-mediated neurogenic activation may converge to produce persistent burning symptoms. This hypothesized effect, though not yet conclusively proven, provides a unifying explanation for the delayed onset and chronicity of symptoms in susceptible individuals. Given the global reliance on PFMs [17,21,22], especially in resource-limited settings, systematically addressing this complication is essential to raise clinical awareness, improve early detection, and guide safer restorative material selection.

In long-serving PFMs, nickel-containing Ni–Cr frameworks are most likely to lower the threshold for the Nallan–Nickel effect once porcelain thins or microfractures. Disruption of the passive film permits slow, persistent nickel release at the saliva–mucosa interface, maintaining a small pool of bioavailable Ni^2+^ that can drive epithelial stress and neuro-immune activation. Co-Cr removes the nickel component but can still accelerate corrosion galvanically when coupled to dissimilar metals (e.g., amalgam, high-noble posts, titanium). High-noble (Au-Pt-Pd) and titanium frameworks exhibit more stable passivation with lower galvanic drive, while all-ceramic options eliminate metal-ion exposure entirely. Clinically, Table 1 supports prioritizing all-ceramic or high-noble replacements when Nallan–Nickel Effect is suspected, avoiding mixed-metal couples in the same quadrant, and polishing/temporarily coating any unavoidable exposed metal until definitive treatment.

## 2. Clinical Concern: Long-Term Wear and Symptom Development

Despite their advantages, PFMs are not without drawbacks [28]. Over time, the porcelain layer may undergo gradual wear, chipping, or even fracture, especially in patients with parafunctional habits or heavy occlusal loads. As the veneering porcelain thins, the underlying metallic substructure becomes increasingly exposed to the oral environment. This prolonged exposure sets the stage for potential biological complications [29,30,31].

One notable clinical observation is the emergence of burning sensations in the oral cavity after years of porcelain-fused-to-metal (PFM) crown service. The mechanism behind this phenomenon is plausibly linked to the release of metal ions from the exposed substructure [32,33]. When the veneering porcelain wears down, the underlying metallic framework comes into direct contact with saliva, dietary acids, and microbial metabolites. This interaction accelerates the corrosion of base-metal alloys such as nickel–chromium or cobalt–chromium, leading to the release of ions such as Ni^2+^, Co^2+^, and Pd^2+^ [29,30,34,35]. These ions may penetrate oral mucosa, irritate epithelial and subepithelial tissues, and exert cytotoxic effects. Nickel in particular is known to impair keratinocyte and fibroblast function, causing low-grade inflammation and delayed healing. In cases where multiple types of metal are present intraorally, galvanic currents may develop, further enhancing corrosion and worsening ion release [36,37].

The burning sensation experienced by patients is not only due to direct irritation but also involves neurogenic sensitization [38]. Importantly, burning mouth symptoms and lichenoid lesions in dental practice can arise from a variety of etiologies. Potential contributors include adverse reactions to metallic ions, hypersensitivity to restorative materials, microbial biofilm changes, autoimmune mucosal diseases such as lichen planus, drug-related lichenoid reactions, systemic conditions including diabetes or xerostomia, and functional pain disorders such as primary Burning Mouth Syndrome (BMS) [39]. Despite careful evaluation, a substantial proportion of cases remain idiopathic, reflecting the complexity of overlapping biological, systemic, and neurological influences. This diversity of possible causes underscores the central challenge in clinical dentistry: distinguishing material-related triggers such as the hypothesized Nallan–Nickel effect from other local or systemic factors.

Metal ions are capable of activating transient receptor potential (TRP) channels, notably TRPV1, which are key mediators of noxious stimuli perception. Their activation lowers the pain threshold, creating persistent burning sensations [40]. At the same time, inflammatory mediators released as a result of corrosion—such as TNF-α, IL-1β, and prostaglandin E2, further sensitize nociceptors in the oral mucosa, amplifying pain signaling. With prolonged stimulation, both peripheral hyperalgesia and central sensitization may develop, similar to the mechanisms proposed in primary burning mouth syndrome, thereby explaining the chronicity of symptoms in some patients [41,42].

Another important factor is hypersensitivity and immunological reactivity. Nickel, palladium, and cobalt, which are frequently used in dental alloys, are well-recognized sensitizers in dentistry. With long-term exposure, some individuals develop type IV hypersensitivity, a T-cell mediated reaction [43,44]. Once sensitized, even minimal ion release can provoke exaggerated responses characterized by burning sensations, erythema, or lichenoid striations [7,43]. In many cases, this manifests as oral lichenoid contact reactions, which are clinically and histologically similar to oral lichen planus [45,46]. The proposed mechanism involves haptenization, where metal ions bind to host proteins and alter keratinocyte antigens, thereby triggering cytotoxic T-cell–driven immune damage. This process is more pronounced in older patients whose mucosal immunity is dysregulated, making them more vulnerable to hypersensitivity responses after chronic exposure [47,48].

The delayed onset of symptoms observed in these cases is consistent with a cumulative, dose-dependent, and time-dependent process. At first, the porcelain veneer acts as a protective barrier, shielding the oral environment from the underlying alloy [49,50]. Over years of occlusal wear, chipping or microfractures can occur; however, saliva gains access to the porcelain–metal interface, which enhances corrosion. Gradually, as the load of released ions increases, the biological tolerance threshold of the oral mucosa may be exceeded, leading to the onset of symptomatic burning or the development of lichenoid lesions [36,51].

## 3. The Nallan–Nickel Effect

We hypothesize that the most distinctive and biologically plausible trigger for burning in long-term PFM wearers is the chronic release of Ni^2+^ from the underlying substructure once the porcelain veneer has thinned or fractured. Nickel has long been recognized as the most frequent sensitizer in dentistry, and even at relatively low concentrations—estimated around 5–10 μg/L in saliva [52], it may act as a hapten, binding to epithelial proteins, altering keratinocyte antigens, and initiating T-cell–mediated hypersensitivity [53].

The cited threshold of 5–10 µg/L in saliva was originally reported in studies on orthodontic appliances and therefore should not be interpreted as a definitive cutoff for patients with single PFM crowns [52]. Instead, it is presented here as a hypothetical benchmark to illustrate the plausibility of a threshold-dependent response. This concentration range is consistent with sensitization data from patch testing, where nickel reactivity is observed at very low exposure levels in susceptible individuals. Moreover, in vitro studies demonstrate that neuronal TRPV1/TRPA1 channels can be activated at micromolar nickel concentrations, supporting the possibility that similar levels in saliva or crevicular fluid could contribute to epithelial stress, immune activation, and neurogenic sensitization [53]. Taken together, the 5–10 µg/L value should be understood as a conceptual anchor for the Nallan–Nickel effect hypothesis rather than a clinically validated threshold in the context of PFM restorations.

Beyond immune activation, nickel ions exert direct cytotoxic effects on keratinocytes and fibroblasts, impairing mucosal repair and promoting a state of low-grade inflammation [54]. Importantly, Ni^2+^ is also capable of activating TRPV1 channels, which are critical mediators of heat and nociceptive signaling [55,56]. Their activation reduces the pain threshold of oral mucosal nociceptors, thereby producing a unique burning sensation rather than nonspecific soreness or ulceration [57]. Other alloy constituents such as cobalt or palladium may contribute to hypersensitivity or lichenoid reactions, but the burning phenotype appears to be most uniquely linked to nickel exposure [58]. We therefore suggest the concept of the Nallan–Nickel effect as a threshold-dependent hypothesis, whereby cumulative nickel ion release may surpass the mucosa’s biological tolerance, initiating immune sensitization and neurogenic activation that could manifest as persistent oral burning. While not yet definitively proven, this hypothesis offers a coherent framework to explain the delayed yet characteristic onset of burning sensations in long-term PFM crown wearers and highlights nickel release as the key determinant in this clinical presentation.

Porcelain wear creates a crevice environment at the porcelain–metal interface where oxygen tension, chloride, and organic acids fluctuate [59]. In base-metal PFMs the protective Cr_2_O_3_ film passivates intermittently; microcracks and low pH (dietary acids, plaque lactic acid) drive film breakdown and mixed active–passive dissolution [60]. Galvanic microcells form between phases in the alloy and with other intraoral metals, increasing local anodic current density and preferential nickel dissolution [61]. In saliva, Ni^2+^ exists as aquo-complexes and as complexes with chloride, bicarbonate, thiocyanate, and thiol-rich proteins (albumin, histatins, cystatines) [62]. Protein binding prolongs mucosal residence while still allowing slow ligand exchange at epithelial surfaces, maintaining a small but persistent pool of bioavailable Ni^2+^—the exposure mode most compatible with delayed symptom onset in long-serving PFMs [63].

At the epithelial barrier, Ni^2+^ can enter keratinocytes through DMT-1/ZIP family transporters and nonspecific Ca^2+^ channels, and it competes at Mg^2+^/Zn^2+^ enzyme sites, disturbing cytoskeletal dynamics and tight-junction scaffolding (ZO-1/occludin), which increases paracellular permeability and facilitates deeper ion penetration [64]. Intracellular nickel perturbs mitochondria (ΔΨm loss, ATP fall), increases ROS, and shifts redox signaling toward Nrf2 and MAPK (p38/JNK/ERK) activation [65]. Although nickel is weakly redox-active, it indirectly amplifies oxidative stress via mitochondrial leakage and by inhibiting antioxidant enzymes, generating 4-HNE and related lipid aldehydes that are potent TRPA1/TRPV1 sensitizers in peripheral nociceptors [66].

Innate immune sensing provides a second amplifier. Human TLR4 can be directly engaged by Ni^2+^ (via histidine-dependent coordination in the TLR4/MD-2 complex), producing rapid NF-κB activation in dendritic cells and keratinocytes, with IL-1β/IL-6/TNF-α release and up-regulation of costimulatory molecules [67]. Concurrently, lysosomal stress from Ni^2+^ uptake triggers NLRP3 inflammasome assembly, yielding caspase-1–dependent IL-1β/IL-18 [68,69]. These cytokines both lower nociceptor thresholds and license robust antigen presentation [70]. On this background, nickel acts as a classical contact hapten, it coordinates with sulfhydryl/imidazole groups on self-proteins, creating neo-epitopes that bind MHC I/II and polarize Th1/Th17 responses. The resulting cytokine milieu (IFN-γ/IL-17A) drives lichenoid interface mucositis and sustains neurogenic inflammation [71].

The neuro-sensory component closes the loop. Ni^2+^ and the associated oxidative/inflammatory mediators sensitize TRPV1 and TRPA1 on peptidergic afferents; acidification and PKC/PKA signaling further potentiate channel gating [72]. Activated fibers release substance P and CGRP, causing mast-cell degranulation (histamine, tryptase) and microvascular leakage, each of which again sensitizes TRP channels and P2X_3_ purinoceptors [73]. Nerve Growth Factor (NGF) induced by epithelial stress promotes peripheral sprouting, converting intermittent irritation into peripheral hyperalgesia and, with time, central wind-up. This neuro-immune crosstalk explains why the dominant patient-reported phenotype is burning rather than dull pain or purely erosive lesions [74].

Nickel’s epigenetic footprint likely prolongs susceptibility. By displacing Fe^2+^ in JmjC histone demethylases and TET dioxygenases, Ni^2+^ favors H3K9 hypermethylation and DNA hypermethylation, stabilizing pro-inflammatory transcriptional programs; it also inhibits prolyl-hydroxylases, stabilizing HIF-1α, which augments nociceptor sensitivity and vascular responses [75]. Together, these changes create a tissue “memory” that makes later, smaller nickel exposures clinically expressive.

The oral ecosystem may intensify exposure. Several plaque bacteria express Ni-dependent ureases; locally elevated nickel can upregulate urease activity, generating ammonia that intermittently raises pH and re-passivates the surface—ironically reducing visual corrosion while sustaining slow Ni^2+^ flux [76]. Sulfur metabolites (H_2_S, thiols) from anaerobes chelate nickel, keeping it soluble; chloride and fluoride at low pH destabilize passive films, especially with acidulated phosphate fluoride products [77]. Low salivary flow concentrates ions, and dissimilar metals create persistent galvanic currents. These factors collectively modulate the effective dose-time integral that determines symptom risk [78].

Within this framework, the Nallan–Nickel effect is the point at which the product of (i) nickel release rate from the restoration, (ii) mucosal exposure time and permeability, and (iii) host neuro-immune sensitivity exceeds an individual tolerance boundary, tipping the system from compensated irritation to self-sustaining burning. The boundary is lowered by prior nickel sensitization, TRPV1 gain-of-function variants, aging-related barrier thinning, and inflammasome-prone genotypes; it is raised by intact saliva, neutral pH, and absence of galvanic couples. The model predicts that a similar mean salivary nickel concentration can be asymptomatic in one patient yet symptomatic in another if permeability or sensitivity terms differ—capturing the clinic’s heterogeneity without abandoning a threshold mechanism.

**Figure 1:**
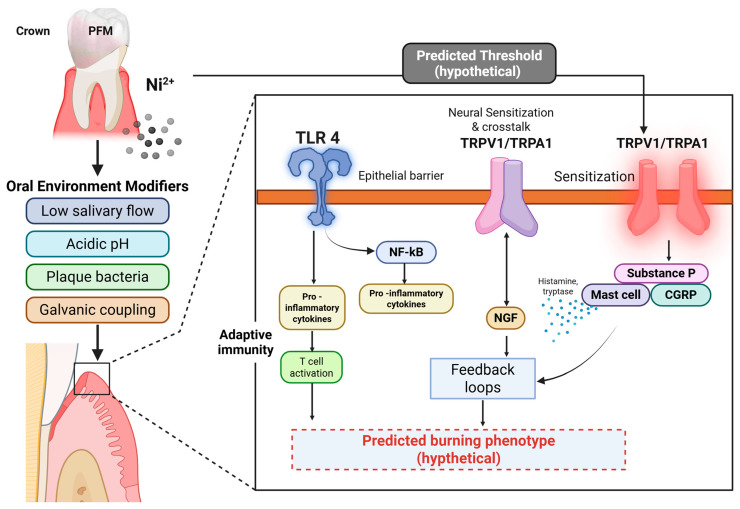
Proposed mechanistic model linking nickel ion (Ni^2+^) release from porcelain-fused-to-metal (PFM) restorations to neural sensitization and burning mouth–like symptoms (the Nallan–Nickel effect). Nickel ions liberated under adverse oral conditions—such as low salivary flow, acidic pH, bacterial plaque, or galvanic coupling—activate toll-like receptor 4 (TLR4) at the epithelial interface. This activation triggers NF-κB–mediated transcription of pro-inflammatory cytokines and promotes T-cell activation, amplifying local immune responses. Released cytokines and nerve growth factor (NGF) engage transient receptor potential channels TRPV1 and TRPA1, producing cross-talk and sensitization of peripheral nociceptors. Mast cell degranulation releases histamine and tryptase, while neuropeptides (Substance P, CGRP) sustain neurogenic inflammation and feedback loops. Together, these mechanisms lower the predicted threshold for neural activation, leading to a hypothetical burning phenotype under sustained exposure conditions. Created with Biorender.com.

Symptomatic PFM wearers expected to show a higher free (unbound) Ni^2+^ fraction in saliva relative to total nickel, increased salivary IL-1β/IL-6, 8-isoprostane, NGF, SP/CGRP, and lowered GSH: GSSG. Buccal biopsies adjacent to exposed PFMs would be expected to exhibit TLR4 and TRPV1 up-regulation, NLRP3 components, and tight-junction disarray. In vitro, conditioned media from corroding Ni-Cr coupons at pH 5.5 should sensitize human oral keratinocytes and trigeminal neurons; TRPV1/TRPA1 antagonists or TLR4 blockade should attenuate this effect. These mechanistic layers—electrochemical release and speciation, epithelial entry and barrier loosening, innate/adaptive immune activation, neurogenic sensitization, and epigenetic locking provide a biologically coherent, threshold-based account of nickel’s role in PFM-associated burning, and they give the Nallan–Nickel effect both explanatory power and clear paths for validation (Figure 1).

Another important long-term concern with porcelain-fused-to-metal restorations is the gradual appearance of oral lichenoid lesions in the adjacent mucosa. A biologically plausible explanation is that, as the porcelain veneer wears down, the underlying metal framework becomes exposed to the oral environment, leading to slow but continuous corrosion and the release of metal ions such as nickel, palladium, or cobalt. These ions can bind to epithelial proteins and alter their surface antigens, effectively turning normal host cells into perceived foreign targets [79]. The immune system, particularly cytotoxic T-lymphocytes, responds by attacking these altered cells, resulting in a localized, cell-mediated hypersensitivity reaction [80]. Clinically, this presents as white striations, erythema, or ulcerations closely resembling oral lichen planus. What makes this reaction distinctive is its chronicity and close topographic relationship with the metal-containing restoration, supporting the notion of a contact-induced lichenoid reaction rather than idiopathic lichen planus [81]. Over time, the persistence of this immune response may not only cause discomfort but also poses a small risk of malignant transformation, which underscores the importance of recognizing the potential biological trade-offs of long-term metal exposure in otherwise durable and cost-effective PFM restorations.

Systemic considerations. Although this Perspective centers on local mucosal pathways, nickel released from compromised PFMs may also contribute to systemic exposure. Swallowed Ni^2+^ can undergo gastrointestinal absorption and enter the bloodstream bound to proteins, with potential distant effects reported in sensitized individuals (e.g., cutaneous flares) and cytokine modulation at higher exposures [82]. While the quantities expected from a single PFM crown are likely far below occupational or dietary peaks, chronic, low-level absorption could act as an adjunctive amplifier of immune tone in predisposed patients or exacerbate comorbidities characterized by systemic inflammation. These points do not alter the local, hypothesis-driven nature of the Nallan–Nickel effect; rather, they place it within nickel’s broader toxicological profile and support cautious interpretation pending direct patient-level data.

## 4. Timeframe for Development of Burning Sensations or Lichenoid Lesions

The onset of burning or lichenoid reactions with PFMs is typically delayed, emerging years after placement. Early in service, the porcelain veneer acts as a barrier. With time, microfractures/chipping expose the alloy; progression then depends on alloy type, corrosion rate, and oral environment (salivary pH, dissimilar metals, diet, hygiene) [83,84,85,86].

A systematic review demonstrated that fixed orthodontic appliances significantly increase salivary nickel and chromium ion levels, with peaks typically occurring within 3–6 months after placement, followed by a gradual decline, though never to zero [87]. These fluctuations were attributed to electrochemical corrosion, mastication-related friction, and dietary or salivary influences [87]. Importantly, even though the concentrations did not usually reach toxic thresholds, the release of nickel and chromium ions was associated with hypersensitivity reactions, burning sensations, and mucosal changes in susceptible individuals [87].

This evidence dovetails directly with clinical observations in long-term PFM crown wearers, where the porcelain veneer gradually wears away, exposing the underlying metallic framework. Much like orthodontic brackets, these metal alloys undergo progressive corrosion, releasing nickel, chromium, and other ions into the oral cavity. The review strengthens this hypothesis by confirming that ion release is not just theoretical but measurable in saliva, and it correlates with clinical symptoms such as burning mouth, metallic taste, mucosal erythema, and lichenoid lesions [87].

The difference in timing between orthodontic appliances and PFMs can be plausibly explained by the nature of exposure. Orthodontic appliances present metallic surfaces that are in direct contact with saliva from the outset, resulting in an early surge of ion release that peak within months before partially stabilizing through oxide layer formation [88]. In contrast, PFMs are initially protected by their porcelain coating, which acts as a barrier to salivary contact. Only after years of occlusal wear, microfractures, or chipping does the underlying metal become exposed, initiating slow but persistent corrosion [89]. This leads to a chronic, low-grade release of nickel and chromium ions that accumulates over time [83,88]. Once ion concentrations surpass an individual’s mucosal or immunological tolerance threshold, symptoms such as burning sensations or lichenoid changes become clinically apparent. Moreover, aging-related thinning of the oral mucosa and immune dysregulation further increase susceptibility in long-term wearers [90].

Thus, while orthodontic appliances show a short- to medium-term surge in ion release, PFM crowns generate a delayed but continuous exposure that explains the late onset of burning mouth and lichenoid reactions in many patients. Taken together, both lines of evidence highlight a shared biological mechanism—metal ion penetration of mucosa, activation of epithelial and dendritic cells, and T-cell–mediated immune responses—that underlies the development of hypersensitivity and contact lichenoid reactions in susceptible individuals.

## 5. Individual Susceptibility

Not all patients who have long-standing PFM crowns develop burning sensations or lichenoid reactions. Susceptibility depends on several biological and environmental factors:

1. Allergic Predisposition: Patients with a known sensitivity to nickel, palladium, or cobalt are at higher risk. Many cases of oral lichenoid contact reactions are essentially delayed hypersensitivity phenomena in predisposed individuals [91].
2. Mucosal and Immune Status: Older individuals often show reduced mucosal barrier function and altered immune regulation, making them more vulnerable to irritation and hypersensitivity [92].
3. Genetic and Epigenetic Factors: Variability in pain receptor expression (e.g., TRPV1 polymorphisms) or immune response genes may explain why some individuals develop neurogenic burning sensations while others remain asymptomatic despite similar exposures [93].
4. Oral Environment: Low salivary flow, acidic oral pH, and the presence of dissimilar metals (which induce galvanic currents) all increase corrosion and ion release, thereby raising the risk of symptoms [78].
5. Duration of Exposure: The longer the crown remains in service after porcelain breakdown, the greater the cumulative release of ions and the higher the risk of symptomatic manifestation [94].

## 6. Management of Symptoms

- When patients present with burning sensations or lichenoid lesions associated with long-term PFM crowns, the first step is careful diagnosis. A thorough history and examination should be undertaken to establish a temporal link between the onset of symptoms and the restoration. Other systemic causes of burning mouth syndrome, such as endocrine, neurological, or psychogenic disorders, should also be excluded. Patch testing for nickel, cobalt, or palladium hypersensitivity can help confirm immunological involvement [95].
- Once a prosthesis-related cause is suspected, the definitive management involves the removal or replacement of the offending PFM crown. Metal-free restorations, particularly zirconia or lithium disilicate crowns, are the preferred substitutes because they eliminate the source of ion release while providing good esthetics and strength. In situations where financial constraints or clinical limitations necessitate continued use of PFMs, selecting high-noble alloys with a higher content of gold or platinum is advisable, as these materials exhibit significantly lower corrosion rates and better biocompatibility [96].
- For patients with active mucosal lesions or burning discomfort, symptomatic relief measures may be required until replacement is carried out. Topical corticosteroids or calcineurin inhibitors (e.g., tacrolimus) can reduce lichenoid inflammation, while topical anesthetics, clonazepam rinses, or capsaicin rinses may help attenuate burning sensations through desensitization of oral nociceptors. Strict oral hygiene maintenance, reduction in parafunctional habits, and avoidance of acidic foods or alcohol-containing mouth rinses further support mucosal healing. Importantly, patients with lichenoid lesions should be kept under regular surveillance, as these lesions carry a small but real risk of malignant transformation [97,98].

From the standpoint of the Nallan hypothesis, modifiable risk factors can be envisioned as operating along a threshold-raising strategy, where immediate measures are aimed at reducing local triggers while longer-term solutions focus on definitive replacement of the offending material. For example, porcelain thinning, microcracks, or the presence of mixed-metal galvanic pairs may lower the hypothesized nickel exposure threshold. In such situations, temporary measures such as smoothing and polishing exposed margins, using neutral rather than acidulated fluoride agents, or applying protective surface coatings may help stabilize the oral environment until replacement with nickel-free, high-noble, or all-ceramic restorations can be pursued [99].

Other influences—such as acidic dietary products, low salivary flow, or parafunctional activity—are also likely to shape nickel bioavailability and material degradation. Acidic conditions increase the proportion of free ions, reduced salivary clearance concentrates them, and excessive occlusal loading accelerates surface failure. Each of these contexts invites targeted, practical responses: dietary counseling, saliva-supportive interventions, or occlusal protection [100].

The guiding perspective is that short-term strategies may buffer against nickel release and galvanic effects, but the ultimate test of the Nallan hypothesis lies in the patient’s clinical response once the suspected source is definitively eliminated. If symptoms resolve after material change, this observation strengthens the plausibility of the proposed mechanism, while ongoing symptoms would invite reconsideration of other contributing factors.

## 7. Other Potential Triggers and Confounders

While nickel is emphasized as the primary suspect in the proposed Nallan–Nickel effect, it is important to recognize that other components of PFM crowns and associated factors may also contribute to adverse outcomes. Cobalt and palladium, both present in commonly used base-metal alloys, can undergo corrosion and ion release. Although their sensitization profiles differ from nickel, they may interact synergistically with nickel to amplify mucosal irritation or immune activation [101]. In particular, cobalt ions have been implicated in oxidative stress pathways, while palladium has been associated with delayed hypersensitivity reactions in sensitized individuals [102]. Additionally, microbial biofilm changes on roughened or corroded PFM surfaces may exacerbate local inflammation [103]. Altered surface chemistry can favor colonization by sulfur-producing bacteria, which generate volatile sulfur compounds that lower local pH and further accelerate alloy degradation, creating a feed-forward loop that enhances metal ion release [104].

It is also essential to differentiate the Nallan–Nickel effect from confounding clinical conditions. Co-existing BMS, a functional pain disorder, may mimic or overlap with symptoms attributed to PFMs. Likewise, systemic conditions such as diabetes, xerostomia, or nutritional deficiencies may lower mucosal thresholds and potentiate symptom expression [105]. In this Perspective, we emphasize that the Nallan–Nickel effect is a hypothesis-driven model specifically linking PFM-derived nickel exposure to burning and lichenoid lesions, but careful clinical assessment must consider alternative triggers and systemic modifiers to avoid over-attribution.

## 8. Differentiation from Primary Burning Mouth Syndrome and Other Lichenoid Conditions

It is important to distinguish the proposed Nallan–Nickel effect from primary Burning BMS and other causes of oral lichenoid lesions. Primary BMS is a functional pain disorder characterized by neuropathic mechanisms in the absence of identifiable local or systemic triggers [106]. In contrast, the Nallan–Nickel effect is a hypothesis-driven model that attributes burning symptoms to a threshold-dependent, local biological response to nickel ion release from porcelain-fused-to-metal restorations. Thus, while both may present with overlapping burning sensations, Nallan–Nickel effect implies a material-related trigger with potential reversibility upon crown replacement, whereas primary BMS persists independently of restorative factors.

Similarly, oral lichenoid lesions may arise from diverse etiologies such as autoimmune lichen planus, drug reactions, or idiopathic mucosal hypersensitivity [107].

Nallan–Nickel effect-related lichenoid reactions differ in that they are hypothesized to emerge specifically in the context of chronic low-level nickel exposure and local immune activation. Careful clinical evaluation, exclusion of systemic causes, and correlation with dental history are therefore essential to avoid misclassification. This comparison underscores the need for rigorous diagnostic criteria and highlights Nallan–Nickel effect as a proposed, testable mechanism rather than a universal explanation for all burning or lichenoid presentations.

## 9. Limitations of the Hypothesis

While the Nallan–Nickel effect provides a mechanistic framework for understanding burning sensations and lichenoid lesions in long-serving PFM crowns, it remains a hypothesis built on indirect evidence. The current model draws largely from in vitro studies, extrapolated immunological mechanisms, and observations in related conditions such as orthodontic appliances and contact hypersensitivity. Direct clinical studies specifically confirming the Nallan–Nickel effect in symptomatic PFM wearers are lacking.

Another limitation is the variability of nickel release in vivo, which depends on multiple patient-specific and environmental factors such as salivary composition, diet, and the presence of dissimilar metals. These make it difficult to establish uniform thresholds across populations. Furthermore, genetic and epigenetic predispositions, while plausible modifiers of susceptibility, have not been conclusively linked to clinical outcomes in this context.

Finally, most of the proposed pathways—such as TRPV1/TRPA1 sensitization, TLR4 activation, and NLRP3 inflammasome engagement—are supported by mechanistic plausibility but require validation in targeted experimental and translational studies. Until such direct evidence is available, the Nallan–Nickel effect should be regarded as a conceptual model that generates testable predictions rather than an established fact.

## 10. Conclusions

In conclusion, the Nallan–Nickel effect should be regarded as a hypothetical framework rather than a proven mechanism. The model is built primarily on indirect evidence, integrating corrosion studies, immunological pathways, and neurogenic sensitization to explain late-onset burning sensations and lichenoid lesions in long-serving PFM restorations. While this synthesis provides a biologically coherent explanation, its validity remains to be demonstrated in clinical and translational studies.

As emphasized in the section “Limitations of the Hypothesis,” the Nallan–Nickel effect must be interpreted with caution, given the absence of direct patient-level validation and the influence of multiple confounding factors such as salivary flow, galvanic coupling, and genetic predisposition. Accordingly, the model should be understood as a proposal that generates testable predictions rather than as an established clinical fact.

Ultimately, the Nallan–Nickel effect serves as a conceptual roadmap. By reframing PFM-related complications as threshold-governed, neuro-immune processes, the hypothesis invites future researchers to design rigorous mechanistic and clinical investigations. Whether validated or refuted, the Nallan–Nickel effect advances dialogue, stimulates targeted inquiry, and fosters a more precise understanding of biologically adverse outcomes associated with long-term dental restorations.

## Figures and Tables

**Table 1:** Dental alloy families and nickel relevance.

Alloy Family	Contains Nickel?	Typical Use with PFM	Corrosion/Galvanic Considerations	Practical Notes	Ref.
**Ni** **-** **Cr base-metal**	Yes	Common legacy PFM frameworks	Susceptible if porcelain thins; galvanic risk with dissimilar metals	Avoid mixing with Co-Cr or amalgam; polish exposed areas; consider replacement	[23]
**Co-Cr base-metal**	No (nickel-free)	PFM frameworks, RPDs	Generally passive oxide, but galvanic vs. other metals	Prefer over Ni-Cr in sensitized patients; avoid mixed-metal couples	[24]
**High noble (Au-Pt-Pd)**	Trace/none (formulation dependent)	Premium PFM	Good corrosion resistance; lower galvanic risk	Safer replacement option for nickel-sensitive patients; all-ceramic also viable	[25]
**Titanium (cp Ti)**	No	Implant abutments/frames	Excellent corrosion resistance; possible galvanic with other metals	Insulate from base-metals; avoid acidulated fluoride agents	[26]
**All-ceramic (zirconia, lithium disilicate)**	No metal	Full-coverage crowns/veneers	No corrosion	Preferred definitive option when Nallan–Nickel Effect is suspected	[27]
